# The Influence of Copolymer Composition on PLGA/nHA Scaffolds’ Cytotoxicity and In Vitro Degradation

**DOI:** 10.3390/nano7070173

**Published:** 2017-07-06

**Authors:** Esperanza Díaz, Igor Puerto, Silvie Ribeiro, Senentxu Lanceros-Mendez, José Manuel Barandiarán

**Affiliations:** 1 Departamento de Ingeniería Minera, Metalúrgica y Ciencia de Materiales, Universidad del País Vasco (UPV/EHU), 48920 Portugalete, Spain; igor.puerto@ehu.es; 2 BCMaterials, Parque Científico y Tecnológico de Bizkaia, 48160 Derio, Spain; senentxu.lanceros@bcmaterials.net (S.L.-M.); manu@bcmaterials.net (J.M.B.); 3 Centro/Departamento de Física, Universidade do Minho, 4710-057 Braga, Portugal; sylvieribeiro88@gmail.com; 4 IKERBASQUE, Basque Foundation for Science, 48013 Bilbao, Spain

**Keywords:** scaffolds, PLGA, nHA, cytotoxicity, in vitro degradation

## Abstract

The influence of copolymer composition on Poly(Lactide-*co*-Glycolide)/nanohydroxyapatite (PLGA/nHA) composite scaffolds is studied in the context of bone tissue engineering and regenerative medicine. The composite scaffolds are fabricated by thermally-induced phase separation and the effect of bioactive nanoparticles on their in vitro degradation in phosphate-buffered solution at 37 °C is analyzed over eight weeks. The indirect cytotoxicity evaluation of the samples followed an adaptation of the ISO 10993-5 standard test method. Based on the measurement of their molecular weight, molar mass, pH, water absorption and dimensions, the porous scaffolds of PLGA with a lower lactide/glycolide (LA/GA) molar ratio degraded faster due to their higher hydrophilicity. All of the samples without and with HA are not cytotoxic, demonstrating their potential for tissue engineering applications.

## 1. Introduction

The manufacture of suitable scaffolds is of the utmost importance in tissue engineering applications. The porosity is essential for their functionality. These structures provide an initial mechanical support and three-dimensional template, facilitating cell adhesion, proliferation and differentiation and the transport of nutrients and metabolic wastes, until the regenerated tissue is structurally stabilized [1,2,3].

These scaffolds have to fulfill a series of structural and chemical requirements for tissue engineering applications. Not only must they be biocompatible, but they must in most cases also replace living tissues capable of growth regeneration and repair, with physical properties that are the consequence of evolutionary optimization over millions of years [4].

A large array of biocompatible materials has been used for this purpose, among which biodegradable polymers are increasingly widely used in tissue engineering. Poly(lactide), poly(caprolactone), poly(glycolide) and their copolymers have attracted a great deal of interest and have been widely used, because they show properties that are comparable to those of biological tissue with the advantage of predictable and reproducible mechanical behavior and degradation. Poly(lactide-*co*-glycolide) (PLGA) is a very popular biodegradable polymer, which has the approval of the U.S. Food and Drug Administration for human clinical applications and combines good mechanical properties, toughness, excellent processability and adjustable degradation rates. Scaffolds should have an interconnected pore structure and high porosity to facilitate cellular adhesion and diffusion of nutrients to cells. A porous interconnected structure and the mean pore size are required to allow diffusion of waste products out of the scaffolds and to allow efficient binding of a critical number of cells to the scaffolds, respectively. The pores need to be large enough to facilitate cells to migrate into the structure. The mean pore size may vary depending on the cell type [5].

One of the most important aspects in porous scaffold development is the control of scaffold degradation behavior. The scaffold must maintain its mechanical properties and structural integrity during the tissue growing process, but it is supposed to degrade and eventually disappear as soon as its purpose is fulfilled, leaving space for newly-formed tissue. It is currently held that the ideal in vivo degradation rate may be similar or slightly less than the rate of tissue growth [6,7,8].

The degradation of aliphatic polyesters, similar to that of PLGA (poly lactideco-glycolide), occurs due to hydrolysis of their unstable ester bonds. End groups are firstly attacked, producing carboxylic acids (lactic and glycolic acids), which are non-toxic and can be removed from the body through the Krebs cycle, but which can act as a catalyst in the degradation process when trapped in the scaffold matrix, accelerating further degradation of the remaining polymer chains.

Degradation behavior has been found to depend on a huge range of factors related with the intrinsic properties of polymers, such as molecular weight, copolymer composition, crystallinity, crystalline size and chain orientation, as well as sample dimensions and shape, including the structure porosity level and pore size. All of these parameters condition the access of water to ester bonds and can influence the degradation process [9,10,11].

The presence of nanohydroxyapatite (nHA) particles does cause a more irregular morphology, as these particles perturb the crystallization of the solvent and change the patterns of crystal growth forming more irregular crystals in the solvent when the fabrication of the scaffolds was by thermally-induced phase separation (TIPS). Additionally, as demonstrated in previous studies, the addition of some bioactive particles can decrease the rate of degradation. The nanohydroxyapatite particles act as a physical barrier and block the entry of water, causing a decrease in the rate of scaffold degradation [9].

There are other external variables, such as temperature, pH and the buffering capacity of the medium in which the degradation occurs. Additionally, in vivo degradation may be accelerated by enzymatic cellular activity or cell-induced pH changes. Even if in vitro degradation models are unable to simulate all of these conditions, these studies provide important information on the degradation behavior of scaffolding.

In this paper, thermally-induced phase separation (lyophilization) was employed to prepare PLGA scaffolds and PLGA/nHA composite scaffolds using two different copolymers: PLGA 53/47 and PLGA 75/25. The temperature was strictly controlled for a convincing in vitro degradation test, because PLGA mainly degrades via chemical hydrolysis, and its biodegradation can be tested with the Arrhenius equation to determine the activation energy [12]. Changes in molecular weight and physical properties were studied, and we found that not only the additives, such as nHA, but also the copolymer composition might affect the degradation behavior. An indirect cytotoxicity evaluation of the samples was conducted through an adaptation of the ISO 10993-5 standard test method.

## 2. Materials and Methods

### 2.1. Raw Materials

Poly(DL-lactide-*co*-glycolide) (PLGA) copolymers in molar ratios of 53/47 and 75/25, respectively, supplied by PURAC (PURASORB, Gorinchem, The Netherlands, PDLG5004 and PDLG7502, respectively) were purified by dissolution in chloroform. The weight-average relative molecular weight of PLGA 53/47 was Mw = 94,800, Mn = 65,600 with a polydispersity of Mw/Mn = 1.4452 and for PLGA 75/25 was Mw = 86,985 and Mn = 53,533 with a polydispersity of Mw/Mn = 1.6250. These values were determined using gel permeation chromatography (GPC, Perkin Elmer 200, Triad Scientific, Manasquan, NJ, USA) in tetrahydrofuran (THF). GPC was performed with a tetrahydrofuran solvent using a reflective index detector with a Perkin Elmer 200 (Triad Scientific, Manasquan, NJ, USA) as the detector. Calibration was done in accordance with polystyrene standards with a flow rate of 1 mL/min. Nano-hydroxyapatite (nHA) was supplied by Aldrich Chemistry (St. Louis, MO, USA), with a particle size >200 nm and Mw = 502.31 g mL^−1^. 1,4 Dioxane purchased from Panreac p.a. (Barcelona, Spain) was used as the solvent. Phosphate-buffered solution (PBS) in water, supplied by Fluka Analytical (Sigma Aldrich, St. Louis, MO, USA) at a pH of 7.2, was used as the degradation fluid.

### 2.2. Fabrication of Porous Scaffolds

Pure PLGA and PLGA/nHA composite scaffolds of both copolymers were fabricated by thermally-induced phase separation (TIPS) followed by a freeze-drying technique. Briefly, PLGA was dissolved in 1,4 dioxane in a proportion of 2.5% (*w*/*v*), by stirring for 2 h at a temperature of 50 °C. After its complete dissolution, the resultant solution was poured into aluminum molds. At this step, nHA was blended by ultrasonic stirring for 5 min, in proportions of 10%, 30% and 50% of total polymer mass, to form the composite scaffolds. The solutions were frozen and freeze-dried for several days to extract the solvent completely. Foams such as porous scaffolds with a porosity of up to 90% were obtained by this method.

### 2.3. Cytotoxicity Assay

An indirect cytotoxicity evaluation of the samples was conducted with the adaptation of the ISO 10993-5 standard test method.

For the in vitro assays, membranes of 0.1 mg mL^−1^ were cut and sterilized by ultraviolet radiation (UV) for 2 h before cell seeding (1 h each side). Afterwards, the samples were washed 5 times with a phosphate-buffered saline (PBS) solution for 5 min to remove any residual solvent.

Briefly, the extraction media were prepared by immersing the samples (0.1 g mL^−1^) in a 24-well tissue culture polystyrene plate with Dubbecco’s Modified Eagle’s Medium-high glucose (DMEM) (containing 1.0 g L^−1^ glucose (Gibco) supplemented with 10% FBS (Biochrom) and 1% P/S (Biochrom)), at 37 °C in 95% humidified air containing 5% CO_2_ and incubated for 24 h. Twenty percent dimethylsulfoxide (DMSO, Sigma Aldrich) was used as a positive control, and the cell culture medium was employed as a negative control.

At the same time, the MC3T3-E1 pre-osteoblast cell line derived from Mus Musculus (mouse) calvaria were seeded in the 96-well tissue culture polystyrene plate at a density of 3 × 10^4^ cells ml^−1^ and then incubated for 24 h to allow for cell attachment on the plate. After 24 h, the culture medium from the 96-well tissue culture polystyrene plate was removed, and the as-prepared extraction medium was added to the wells (100 μL). Afterward, the cells were incubated for 24 h and 72 h, and at each control point, the evaluation of the cell viability was quantified with a 3-(4,5-dimethylthiazol-2-yl)-2,5-diphenyltetrazolium bromide (MTT) assay.

The MTT assay measures the mitochondrial activity of the cells, which reflects the viable cell number. At each point in time, the medium of every well was removed, and a fresh medium containing 10% MTT solution (stock solution of 5 mg MTT mL^−1^ in PBS; Sigma Aldrich) was added. The viable cells with an active metabolism converted MTT into a purple-colored formazan product. After 2 h of incubation, the MTT crystals were dissolved with DMSO, and the optical density was measured at 570 nm.

All quantitative results were obtained from four replicate samples with controls and were analyzed as the average of viability ± the standard deviation (SD).

The percentage of cell viability was calculated with the following formula:

(1) $$\mathrm{Cell}\;\mathrm{viability}\;\left(\%\right)=\frac{absorbance\;of\;sample}{absorbance\;of\;negative\;control}\times 100$$

### 2.4. In Vitro Degradation

Samples for in vitro degradation were cut into 0.5-cm^2^ rectangular pieces and weighed. The specimens were then placed in identical glass vials containing 10 mL of PBS, totally immersed and incubated in a thermostatic oven at 37 °C, under static conditions. At selected points in time (1, 2, 4, 6 and 8 weeks), the specimens were recovered, carefully wiped to remove surface water and weighed to determine water absorption. The pH change in the degradation medium was determined using a PCE 228 pH meter from PCE Instruments (Alicante, Spain) and corrected by temperature controls. Finally, the samples were dried over 2 weeks to a constant weight that was recorded, in order to determine the weight loss.

### 2.5. Characterization

#### 2.5.1. Water Absorption and Weight Loss

Water absorption and weight loss were evaluated by weighing. The percentage water absorption *W_a_*% was calculated with the following equation:

(2) $$W_{a}\%=\frac{W_{w}-W_{r}}{W_{r}}\times 100$$

where *W_w_* is the weight of the wet/swallow specimen after removing surface water and *W_r_* is the residual weight of a completely dry sample after degradation. Weight loss percentage (*W_L_*%) was estimated with the following equation:

(3) $$W_{L}\%=\frac{W_{0}-W_{r}}{W_{0}}\times 100$$

where the original mass of the sample is designated as *W*_0_.

#### 2.5.2. SEM Analysis

The bulk morphology of the scaffolds was examined using scanning electron microscopy (SEM) (HITACHI S-3400N, HITACHI, Tokyo, Japan). Prior to analysis, the samples were coated with a layer of gold, in a JEL Ion Sputter JFC-1100 (JEOL, Peabody, MA, USA at 1200 V and 5 mA, to avoid sample charging under the electron beam.

#### 2.5.3. DSC Analysis

The thermal characteristics of the polymer were determined using differential scanning calorimeter (DSC TA Instruments, Waters, NC, USA equipped with an intracooler. Approximately 10 mg of polymer were placed in a crimp-sealed DSC hermetic aluminum pan. A nitrogen purge gas was used to prevent oxidation of the samples during the experiments, which were subjected to temperature scans ranging between −20 °C and 200 °C at temperature/time ratios of 10 °C/min.

## 3. Results and Discussion

### 3.1. Cytotoxicity

**Figure 1:**
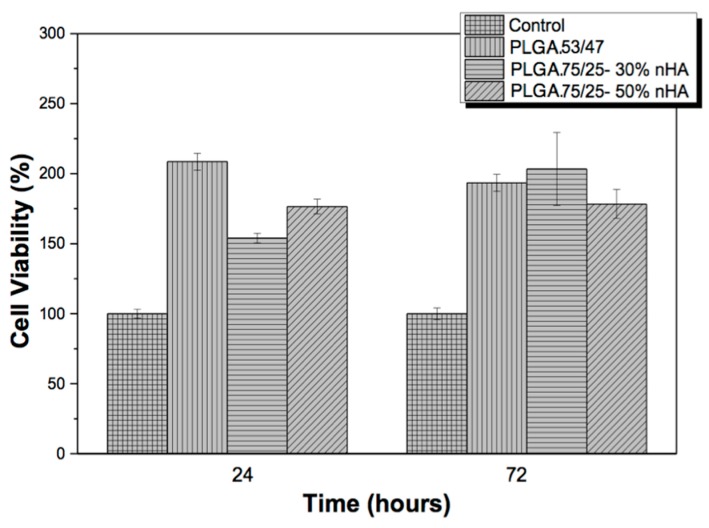
MTT cytotoxicity assays of MC3T3-E1 pre-osteoblast in contact with the as-prepared extraction media exposed to PLGA and PLGA composites with 30 and 50% nHA.

The cytotoxicity of the different PLGA and PLGA/nHA composite samples using two different copolymers, PLGA 53/47 and PLGA 75/25, was evaluated by the MTT assay method against the MC3T3-E1 pre-osteoblast cell line. The results are presented in Figure 1.

Figure 1 shows that none of the samples were cytotoxic after 72 h. According to ISO standard 10993-5, samples are considered cytotoxic when the cell viability reduction is larger than 30%. The incorporation of bioactive reinforcements is considered as a powerful method to improve the properties of polymer [13]. Previous studies reported that these materials were not cytotoxic, and hydroxyapatite improved cell adhesion [14]. In this case, the cell viability of the samples shows values higher than 100%, after 72 h, which can be related to the increased proliferation due to the materials used. In fact, the presence of nHA has been proven to have an inductive effect on the proliferation of the osteoblast cells [15,16].

**Figure 2:**
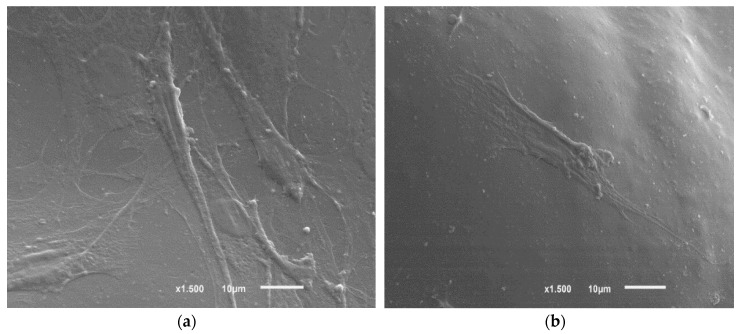
Cell morphology of MC3T3-E1 pre-osteoblasts seeded on PLGA 75/25 (**a**) 10% nHA; and (**b**) 50% nHA samples after three days obtained by SEM. The scale bar is 10 µm for all samples.

In order to determine the suitability of the membranes for tissue engineering and biomedical applications, the cell morphology of cells grown on the PLGA membranes with nHA was assessed with the SEM assay. The representative scanning electron micrographs are shown in Figure 2.

Figure 2 shows the cell morphology of MC3T3-E1 pre-osteoblast cells after three days of culture on the PLGA with nHA samples. Comparing the samples, the cells seem to maintain random arrangement on the membranes. Sheikh et al. demonstrated that the samples with nHA are good candidates for in-bone tissue regeneration because the introduction of these particles showed improvements in hydrophilicity, mechanical properties, viability of osteoblasts and complete formation of bone in in vivo experiments [17].

### 3.2. In Vitro Degradation

Control over degradation kinetics is of vital importance for the manufacture of porous scaffolds. Degradation that is too fast could compromise the mechanical integrity of the scaffold, while if it is too slow, the scaffold would interfere in the correct integration of the regenerated tissue. The scaffold should ideally have a speed of degradation equal to or slightly slower than the growth of the tissue [6,9,18].

#### 3.2.1. Mass Loss and Molecular Weight

The reduction of the molecular weight and the increase of polydispersity are among the first indicators of polymeric degradation processes. The reduction in molecular weight occurred with the breakage of the polymer chains as a consequence of hydrolysis, and the increase in broken chains (shorter and with less molecular weight) increased polydispersity.

The analysis of the samples with GPC, the results of which may be seen in Table 1 and Table 2, permits the calculation of the weight-average molecular weight (Mw), number-average molecular weight (Mn) and polydispersity (I = Mw/Mn). A systematic reduction of the molecular weight is observed, practically from the start of degradation in both systems—PLGA 53/47 PLGA 75/25—under study, and this reduction is more accentuated in the scaffolds that have no bioactive particles. The scaffolds with nHA also underwent a loss of molecular weight, although this was notably lower than in the samples of pure polymer. The presence of nanoparticles had a shock absorber effect on the interface between PLGA and nHA, avoiding the penetration of PBS into the walls of the scaffold and breaking the degradation by neutralizing the catalytic effect of the carbonyl groups that formed when the polymer chains were broken. The concentration of nHA did not appear to be a decisive factor. For example, the reduction of the molecular weight of the samples with PLGA 75/25 was comparable to those with a greater concentration of nanoparticles. In fact, among the samples with nHA, those with 30% nHA underwent a reduction in molecular weight that was slightly higher than the rest. This reduction is due to the presence of modified particles that modify the pores, which is another of the decisive factors in the degradation process.

Comparing the scaffolds fabricated with the two copolymers, we can see that PLGA 53/47 underwent a much faster reduction in molecular weight than PLGA 75/25, so much so that the pure samples of PLGA 53/47 were almost completely degraded within three weeks. Minor losses of molecular weight were experienced in the PLGA (75/25) samples. The Mw is almost always double at the end of the grading period in the 75/25 system, due to the lower content of glycolic acid in the system. A higher content of hydrophilic glycolic acid units in the copolymers facilitates the absorption and diffusion of water and, as a result, hydrolysis [8,19]: the degradation rate therefore increased by as much as:

PLGA 75/25 < PLGA 53/47

**Figure 3:**
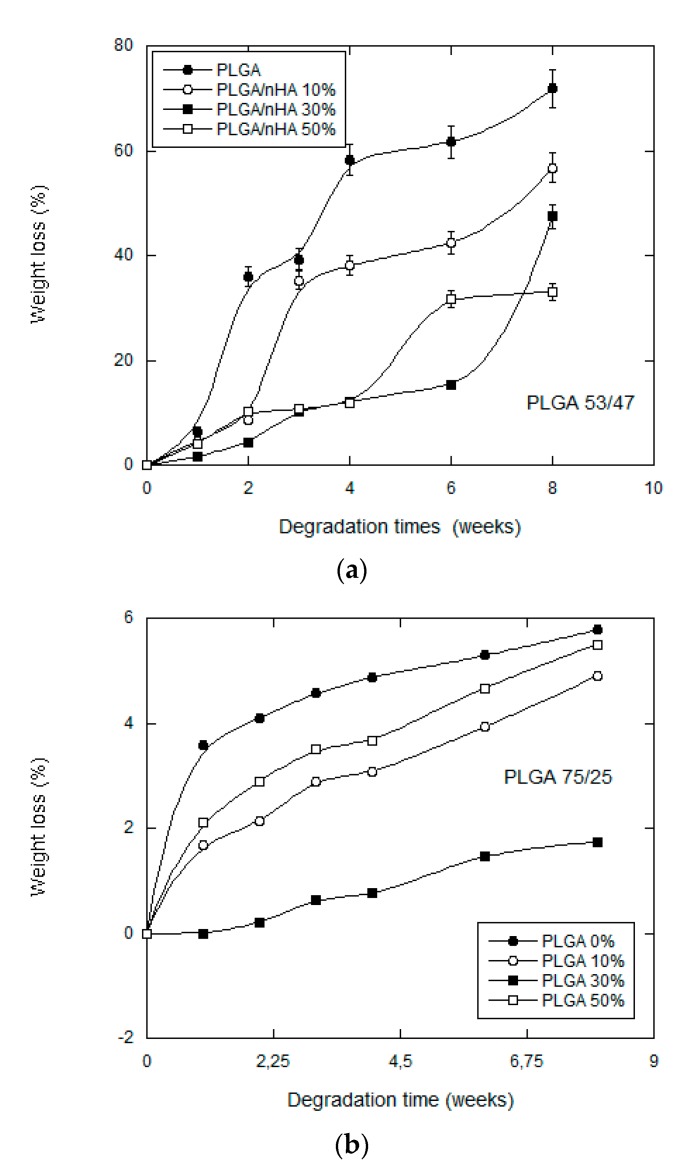
Mass loss of: (**a**) PLGA (53/47)/nHA; and (**b**) PLGA (75/25)/nHA, composite scaffolds against degradation time.

Other researchers [19,20] who conducted studies on degradation observed that the loss of molecular weight in an en bloc degradation mechanism starts as soon as the polymer enters into contact with the water, while the loss of mass will not start immediately and, in any case, not until the polymer reaches a critical molecular mass. In our case (see Figure 3), all of the samples lost mass from the first week of degradation. The samples that lost most mass were those of pure PLGA: the samples of the PLGA 75/25 copolymer losing almost 6% of their original weight at eight weeks and those of the PLGA 53/46 copolymer losing 70% of their original weight.

The scaffolds manufactured with PLGA 53/47 without bioactive particles were almost completely degraded at the end of eight weeks. In much the same way, the samples with nHA also presented a loss in mass, although a much lower one.

The results appear to indicate that the scaffolds of PLGA 75/25 started to become degraded on the surface from the first week, especially those that contained no nHA, subsequently undergoing en bloc degradation, which is the habitual form of degradation for this type of polyester [6].

#### 3.2.2. Water Uptake

**Figure 4:**
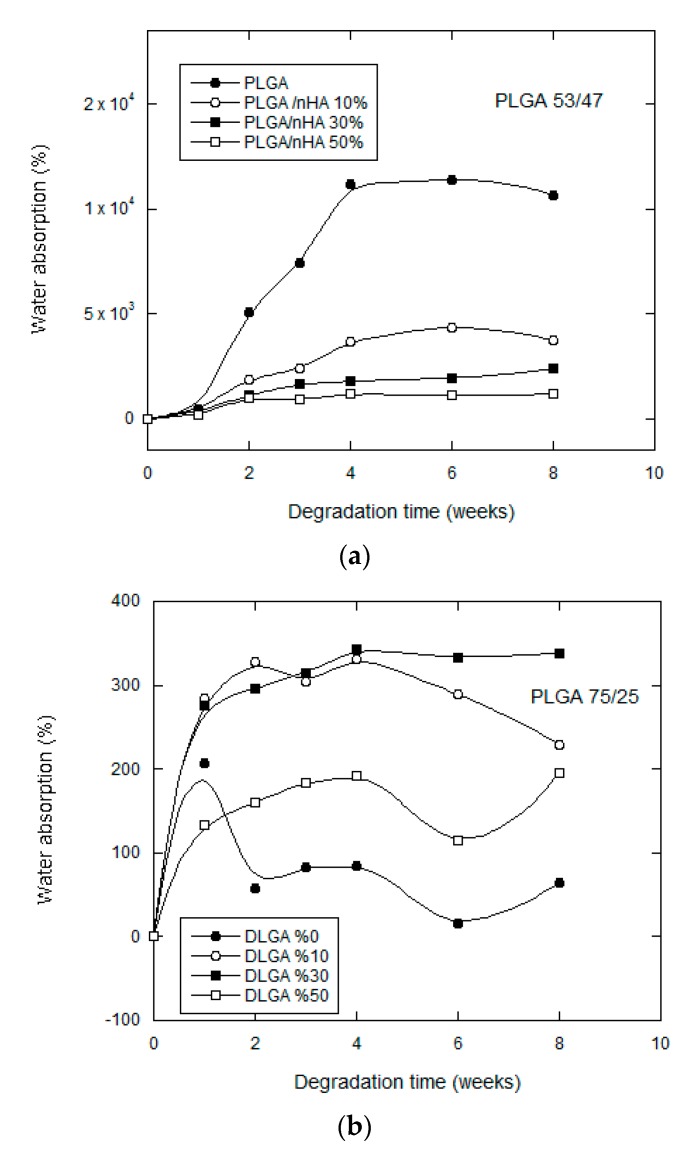
Water absorption for: (**a**) PLGA (53/47)/nHA; and (**b**) PLGA (75/25)/nHA composite scaffolds as a function of degradation time.

We may see that in Figure 4, for both copolymers under study, that there was very significant water uptake in the scaffolds up until the fourth week of in vitro degradation, after which the absorption rate stabilized. In addition, the water uptake of the samples without bioactive particles was far less than the water uptake of the samples that contained bioactive particles. If we compare both copolymers, we see that the samples that absorbed most water corresponded to PLGA75/25, which achieved much higher values than those of PLGA 53/47. PLGA 75/25 contains a higher proportion of lactide in its chain and behaves in a much more hydrophobic way, the water entering the scaffolding at first through its pores, and then, equilibrium is achieved between the absorption of PBS and the dissolution of oligomers, so it is very probable that the part of the chain that degrades first of all is that of the glycolic acid, which is much more hydrophilic than polylactide This all indicates that the degradation mechanism in this case will be mixed, with the predominating influence of en bloc degradation that reduces the molecular weight, accompanied by a surface degradation, which explains the notable loss in weight.

However, the inclusion of nHA modifies the hydrophobic condition of the polymer and favors its absorption in the buffer solution (PBS). This behavior has been observed by other authors [21,22] who concluded that the nHA could accelerate degradation by the hydrolysis of certain polymers when favoring water uptake. In our case, the nHA nanoparticles provoked high levels of water uptake, despite which, degradation of the samples was slower rather than quicker. The nanoparticles in our scaffolds acted as a physical barrier, absorbing the water, but complicating its diffusion towards the walls of the porous scaffold.

The addition of high amounts of nanoparticles modified the morphology. Greater irregularity in the pores provoked by a larger amount of nHA for the samples of PLGA/50 wt% nHA might imply a less uniform distribution and an agglomeration of nHA particles, which formed bigger particles with smaller surface areas. This is why the samples of both copolymers made with a higher content of nanoparticles have a more irregular behavior (see Figure 3 and Figure 4). The distribution of the nanohydroxyapatite plays an important role not only in the water uptake, but also in the degradation process itself.

#### 3.2.3. pH

The pH variations of the degradation medium offer information on the liberation of acidic products as a consequence of the splitting reactions due to hydrolysis. In the same way, these variations in pH can condition the degradation by modifying the conditions of the medium [23].

**Figure 5:**
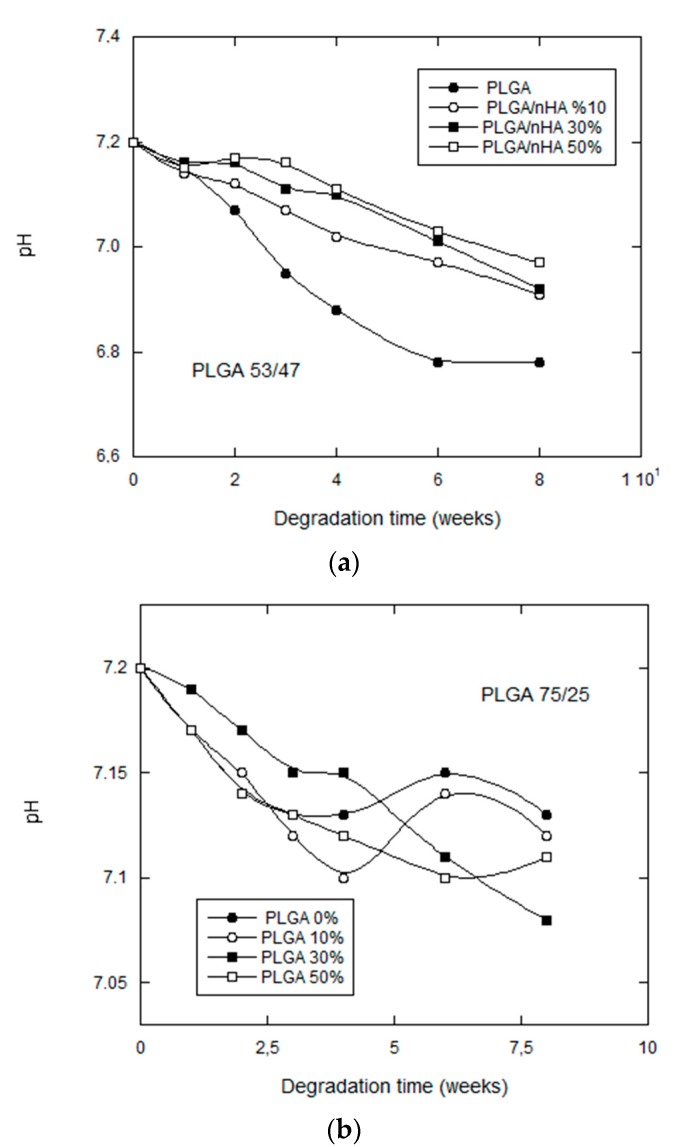
pH change of phosphate-buffered solution for: (**a**) PLGA (53/47)/nHA; and (**b**) PLGA (75/25)/nHA composite scaffolds against degradation time.

The general tendency for all of the samples was a moderate reduction in the pH of the degradation medium, as may be confirmed in Figure 5, except for the scaffolds manufactured with the copolymer PLGA 75/25, which tended to stabilize as from the fourth week of in vitro degradation.

Unlike what took place with PLGA 53/47, the pH of the samples without nHA fell slightly, indicating that they freed a smaller quantity of acid from the medium than the others. Taking into account that the results of GPC indicated that these were the most degraded samples, it is quite possible that they were undergoing a self-catalyzed degradation en bloc [24,25], in which case the acidic products would have been kept inside the scaffold and would not have been freed. At another extreme, we have the samples with 30% nHA, which underwent a greater reduction in their pH, because diffusion mechanisms, due to a higher water uptake, assisted the movement of oligomers into the PBS. The porous scaffolds of 50% nHA might have been expected to have shown a lower pH, due to the effect of the nanoparticles, but such a high quantity of these participles can provoke a tendency to coalescence, as previously observed, before the manufacture of the scaffolds.

#### 3.2.4. Thermal Analysis (DSC)

**Figure 6:**
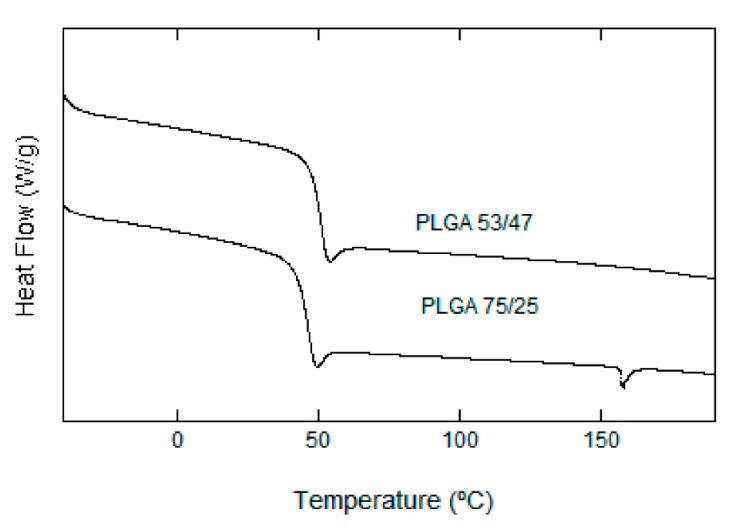
Thermograms of PLGA (53/47) and PLGA (75/25).

The thermal behavior of the was studied by performing two scans from −50 to 200 °C with a heating speed of 10 °C/min and a cooling speed of 20 °C/min. If we compare the thermograms of both PLGA 75/25 and PLGA 53/47 (Figure 6), we can confirm that they both behaved in a similar way; the glass transition temperature (Tg) (first run) of PLGA 53/47 was at 56 °C, while the Tg of PLGA 75/25 took place at around 50 °C; however, the most notable observation was that the semicrystalline PLGA 75/25 showed a small endothermic fusion peak at 170 °C, while PLGA 53/47 was amorphous. This peak was not shown in the subsequent thermograms performed with our scaffolds, undoubtedly due to the thermal treatment used during their manufacturing (a rapid tempering at −62 °C in addition to the degradation tests, performed under the Tg of the material, preventing any possible crystallization later on). The totality of the samples therefore presented an amorphous behavior throughout the degradation process.

The degree of crystallization must therefore significantly influence the degradation behavior. Some researchers have reported that the degradation behavior of amorphous PLGA scaffolds was more suitable for bone tissue engineering, because of more mineralized tissue formation within the matrix [8,13].

The Tgs shown in Table 3 and Table 4 were determined from the DSC curve of the second run. In Table 3, we can see how the Tg temperature increased with the concentration of nHA and with the in vitro degradation for the samples fabricated with PLGA 53/47. On the contrary, no variations of Tg were observed in the samples of PLGA 75/25 (see Table 4).

The effect of the molecular weights of the copolymers on the Tg in the in vitro degradation process may be neglected, because the weights were sufficiently high. The Tg depends mainly on the copolymer composition. A higher GA content in the PLGA results in a higher Tg in the in vitro degradation, because PLGA 53/47 has a higher amorphous content; this behavior is contrary to what has been found by other researchers [6,26,27,28]. Samples that have been fabricated with PLGA 75/25 do not experience a variation in Tg because the degradation process is much slower than those of PLGA 53/47.

#### 3.2.5. FTIR

The absorption spectra of the samples was measured with Fourier transform infrared (FTIR) spectroscopy and an attenuated total reflectance (ATR) accessory, so as not to have to modify the samples, because copolymer dissolution when preparing the films might provoke the presence of non-dissolved nHA.

**Figure 7:**
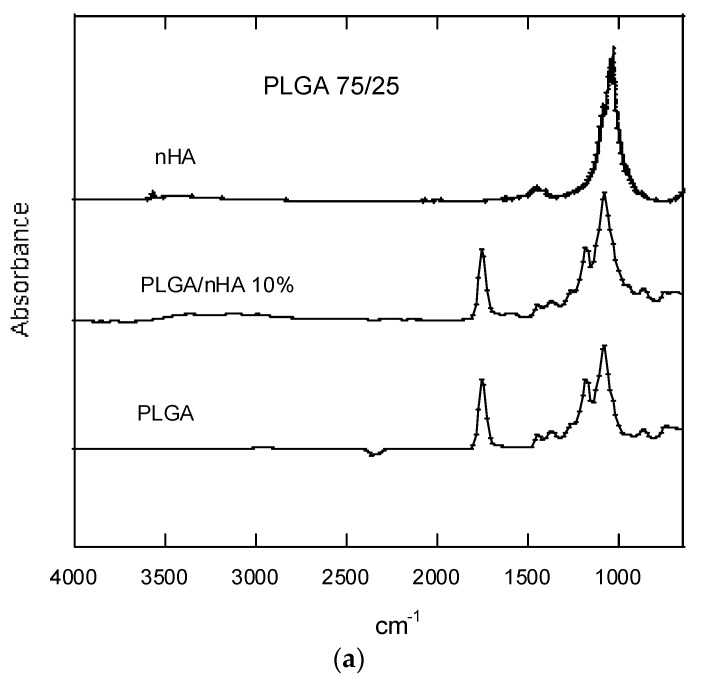

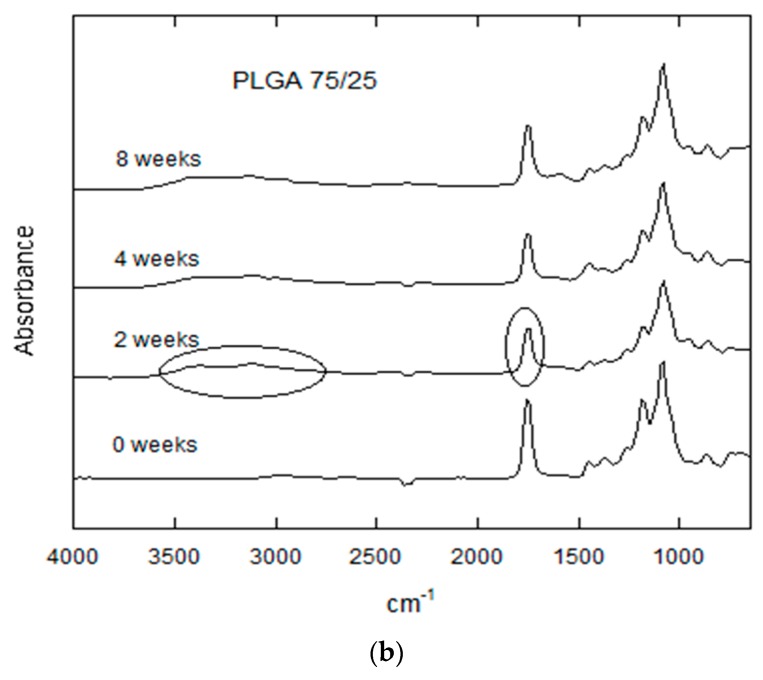
(**a**) FTIR spectra of nHA, PLGA (75/25) and PLGA (75/25)/nHA 10%; (**b**) FTIR spectra of PLGA (75/25) and PLGA (75/25)/nHA 30% after various degradation times.

As we have already seen in other studies completed earlier [23,26,27,28], the presence of nHA will not affect the functional groups that we can observe through FTIR spectroscopy (see Figure 7a).

The degraded scaffolds hardly presented appreciable variations. One of them was the appearance of a broad, but hardly intense band, after the second week of degradation, between 2800 and 3600 cm^−1^, which could be attributed to the stretching vibration of the OH bands of the COOH and OH groups (Figure 7b).

A very prominent absorption band appeared in the 1756-cm^−1^ region corresponding to the ester of the carbonyl group. This band is typical in the copolymers regardless of the type of PLGA.

#### 3.2.6. SEM

The morphology of the scaffolds was studied with scanning electron microscopy (SEM). The particles, included before the lyophilization process, are found uniformly distributed in the polymer matrix. Scaffold porosity exceeded 90%. In previous publications [7,24,28], the research group has reported that when using 1,4 dioxane and the resulting heat treatment in the manufacturing process, the pore structure and morphology were controlled by the solid-liquid phase separation process of the polymer solution. The pore size and the porosity are both attributed to a wall effect instead of a surface area. The morphology of the PLGA and PLGA/nHA composite scaffolds was similar for the samples prepared with the same procedure of fabrications, and they had a highly anisotropic tubular morphology with an internal ladder-like structure.

**Figure 8:**
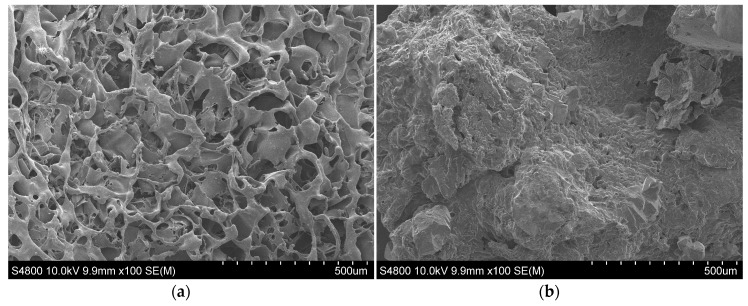

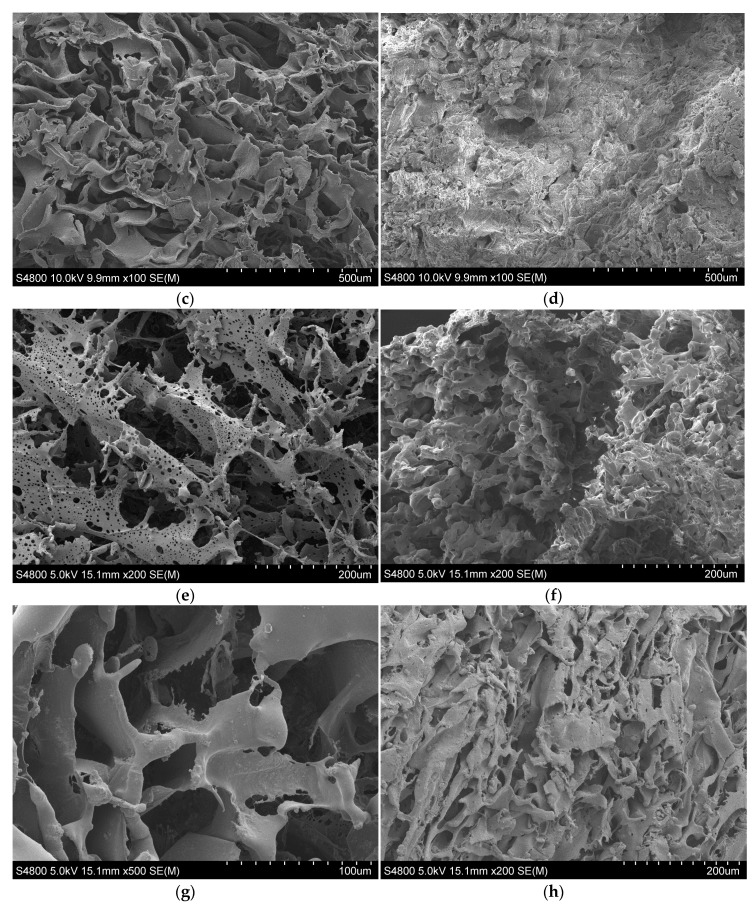
SEM observation of surface morphology of PLGA. (**a**) PLGA (53/47) before degradation; (**b**) PLGA (53/47) after in vitro degradation for three weeks; (**c**) PLGA (53/47)/nHA 50% before degradation; (**d**) PLGA (53/47)/nHA 50% after in vitro degradation for six weeks; (**e**) PLGA (75/25) before degradation; (**f**) PLGA (75/25) after in vitro degradation for eight weeks; (**g**) PLGA (75/25)/nHA 50% before degradation; (**h**) PLGA (75/25)/nHA 50% after in vitro degradation for eight weeks.

PLGA 75/25 produced highly porous and interconnected scaffolds, as may be appreciated in Figure 8, the walls of which presented a high microporosity. PLGA 53/47 presented no porosity in the walls of the pores, and these were thicker than the pores of PLGA 75/25. The addition of nHA for both copolymers had no effect on porosity, but it did reduce the size and the morphology of the pores by interfering in the crystallization process of the dissolvent. As a result of irregular solvent crystal growth, the pores and the structure became irregular (more isotropic). Samples with a low content of nHA had a more porous appearance.

During the in vitro degradation in the porous scaffold of PLGA 75/25, microporosity was disappearing at the same time as the scaffold started to lose its form. Erosion was evident from the first week, and from the fourth week, the structure of the scaffold started to collapse. After the first week of degradation, a change in the surface morphology was appreciated in the porous scaffolds of PLGA 53/47 that went from having a smooth appearance to a more rugged one. This change is without a doubt due to the degradation, which started to free its products, exposing them together with particles of nHA on the walls of the scaffold. The formation of these precipitates on the surface could favor the interaction between material and cell and therefore its biological response; moreover, the speed of degradation of the scaffolds was much quicker than those manufactured with PLGA 75/25. The nanoparticles in both cases appeared to delay the process of in vitro degradation.

In this way, we were able to confirm that the porous scaffolds of PLGA 75/25, because they contained 75% DL-lactide and only 25% glycolic acid, degraded much more slowly (a difference visible to the naked eye), while the samples of PLGA 75/25 were much less swollen and deformed than those of PLGA 53/47. The higher amount of lactide in the chain had a direct influence on the degradation. This tendency has previously been observed by other researchers [9,19].

## 4. Conclusions

Studies of in vitro degradation in a PBS solution of composite scaffolds manufactured with PLGA 75/25, PLGA 53/47 and nHA have allowed us to confirm the influence of the copolymer ratio and the presence of nanohydroxyapatite as moderating elements in the degradation process. The inclusion of nHA modified the hydrophobic condition of the polymer and favored the entry of PBS, despite which, degradation was no quicker in the samples, but quite the contrary. The nanoparticles in our scaffolds acted as a physical barrier absorbing water, but complicating their diffusion towards the walls of the porous scaffolding. Moreover, PLGA 75/25, as it contains 75% DL-lactide and only 25% glycolic acid, degraded much more slowly than the samples of PLGA 53/47, which became much more swollen and deformed. The PLGA with the lowest LA/GA molar ratio degraded faster due to its higher hydrophilicity. The PLGA samples and the corresponding composites with the inclusion of nHA are not cytotoxic. The introduction of hydroxyapatite is a bioactive reinforcement for bone tissue engineering applications.

## Figures and Tables

**Table 1:** Mw, Mn and I as a function of degradation time for PLGA (53/47) and PLGA (53/47)/nHA composite scaffolds.

Degradation Time (Weeks)	PLGA 53/47	
	0% nHA	30% nHA
**Weight-Average Molecular Weight (Mw)**		
0	94,800	94,800
1	29,916	58,670
2	-	35,425
3	890.7	23,106
4	-	23,811
6	-	22,141
8	-	22,936
**Number-Average Molecular Weight (Mn)**		
0	65,600	65,600
1	16,688	33,744
2	-	10,708
3	430	10,831
4	-	11,690
6	-	15,644
8	-	10,923
**Polydispersity (I)**		
0	1.445	1.445
2	1.792	1.738
3	-	3.308
4	2.071	2.133
6	-	1.415
8	-	2.099

**Table 2:** Mw, Mn and I as a function of degradation time for PLGA (75/25) and PLGA (75/25)/nHA composite scaffolds.

Degradation Time (Weeks)	PLGA 75/25			
	0% nHA	10% nHA	30% nHA	50% nHA
**Weight-Average Molecular Weight (Mw)**				
0	86,985	86,985	86,985	86,985
2	73,311	79,604	78,758	79,455
3	72,334	75,392	73,433	78,577
4	66,296	75,368	69,166	72,973
6	56,212	68,749	57,371	66,178
8	39,979	56,101	49,760	58,429
**Number-Average Molecular Weight (Mn)**				
0	53,533	53,533	53,533	53,533
2	46,131	48,388	48,979	43,404
3	42,983	43,884	44,238	45,835
4	39,782	45,490	41,345	41,808
6	33,430	40,419	32,253	38,109
8	19,524	31,412	29,689	33,255
**Polydispersity (I)**				
0	1.650	1.625	1.625	1.625
2	1.589	1.645	1.608	1.830
3	1.682	1.718	1.659	1.714
4	1.666	1.656	1.672	1.745
6	1.681	1.701	1.778	1.736
8	2.047	1.786	1.676	1.757

**Table 3:** Tgs of PLGA (53/47) and PLGA (53/47)/nHA composite scaffolds.

Degradation Time (Weeks)	Tg (°C)			
	nHA 0%	nHA 10%	nHA 30%	nHA 50%
0	18.2	30.2	32.8	28.9
1	44.2	33.7	34.4	30.7
2	N/A	35.9	36.3	33.5
3	N/A	38.4	39.4	38.8
4	N/A	35.9	43.7	43.5
6	N/A	N/A	40.2	40.6
8	N/A	N/A	40.1	40.9

**Table 4:** Tgs of PLGA (75/25) and PLGA (75/25)/nHA composite scaffolds.

Degradation Time (Week)	Tg (°C)			
	nHA 0%	nHA 10%	nHA 30%	nHA 50%
0	51.5	50.4	52.9	51.0
1	52.2	52.8	51.9	51.8
2	51.8	52.1	51.5	53.1
3	52.6	52.2	51.9	52.5
4	52.1	52.4	51.8	52.8
6	52.1	52.1	51.9	52.9
8	51.0	51.9	52.1	52.5
